# Structural DNA nanotechnology at the nexus of next-generation bio-applications: challenges and perspectives

**DOI:** 10.1039/d3na00692a

**Published:** 2023-12-19

**Authors:** Sanjay Kosara, Ramesh Singh, Dhiraj Bhatia

**Affiliations:** a Department of Biological Sciences and Engineering, Indian Institute of Technology Gandhinagar Palaj Gujarat 382355 India; b Department of Mechanical Engineering, Colorado State University Fort Collins CO USA dhiraj.bhatia@iitgn.ac.in ramesh.singh@colostate.edu.in

## Abstract

DNA nanotechnology has significantly progressed in the last four decades, creating nucleic acid structures widely used in various biological applications. The structural flexibility, programmability, and multiform customization of DNA-based nanostructures make them ideal for creating structures of all sizes and shapes and multivalent drug delivery systems. Since then, DNA nanotechnology has advanced significantly, and numerous DNA nanostructures have been used in biology and other scientific disciplines. Despite the progress made in DNA nanotechnology, challenges still need to be addressed before DNA nanostructures can be widely used in biological interfaces. We can open the door for upcoming uses of DNA nanoparticles by tackling these issues and looking into new avenues. The historical development of various DNA nanomaterials has been thoroughly examined in this review, along with the underlying theoretical underpinnings, a summary of their applications in various fields, and an examination of the current roadblocks and potential future directions.

## Abstract 2

The review covered DNA nanotechnology's current use in physical and biomedical fields, its challenges, and future prospects, emphasizing integration with biological systems and enhanced compatibility.

## Graphical abstract

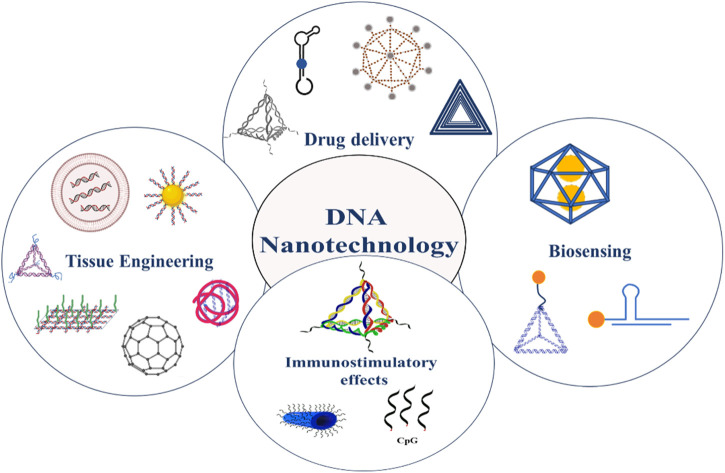

## Introduction

Biomolecular self-assembly is a common technique used by organic material scientists to synthesize functional nano-biomaterials. These abundant materials have unique structures, functions, compatibility with biological systems, and atomic-level tailorable properties.^1,2^ DNA is a biopolymer that stores and carries genetic information in all living systems. Owing to its unique chemical and structural properties, DNA is now being used as a programmable material building block.^3,4^ The dimensions of a DNA duplex are at the nanoscale, making it suitable for nanoscale construction. Structural DNA nanotechnology utilizes designed nucleic acids as building blocks to create thermodynamically stable nanostructures through molecular self-assembly. These nanostructures are developed using Watson–Crick base-pairing interactions between well-designed single-stranded DNA (ssDNA) oligomers and possess highly accurate structural predictability and programmability.^5–8^

Seeman's pioneering works showed the synthesis of DNA nanostructures, which led to the rapid development of DNA-based nanostructures.^9–13^ With the development of different computer-based tools^14–19^ for designing DNA sequences for creating nanostructures of interest, DNA nanotechnology has been effectively proven in the production of several significant nanostructures ranging from linear and two-dimensional to three-dimensional nanodevices in the last four decades.^8^ These nanostructures include branched DNA motifs,^12,20^ tile assemblies,^8,21–23^ origami structures,^24–27^ nanocages^28^ and dynamic nanostructures.^29,30^ DNA nanotechnology has emerged as a promising technology with several advantages over traditional materials, including high storage density, potentially low energy requirements, and long-term stability. The field has seen applications to solve fundamental scientific problems in structural biology, biophysics and medicinal biology.^4^ These applications include tissue engineering,^4,31–34^ immune engineering,^35,36^ drug delivery,^37–45^ disease diagnosis^4,46,47^ and molecular biology tools or biosensors.^45,47,48^ DNA structures have unique properties compared to other biopolymeric materials and nano-nanomaterials. The structure of DNA-based nanomaterials allows for flexibility as every strand can be concatenated or linked with an extended arm. The assembly of DNA frameworks provides a hollow internal space for drug molecules, enabling efficient drug delivery. DNA nanoparticles have a negative charge, which allows for the integration of positively charged substances through electrostatic attraction. They can serve as both construction material building blocks and therapeutics, such as in self-assembled spherical nucleic acids that exhibit high cellular uptake and perform gene knockdown.^49^

DNA nanoparticles have been extensively researched for their impact on cell biological activities, specifically cell proliferation, and their role in tissue regeneration in various tissues, such as bones,^50–53^ cartilages,^54^ blood vessels, nerves,^4,55^ skeletal^34^ and cardiac muscles, the skin and corneal systems. DNA nanocages have been used to stimulate the proliferation and differentiation of stem cells, leading to changes in gene expression and subsequent alterations in cell properties and morphology.^56,57^ This process modifies the cells' sensitivity to different stimuli, ultimately resulting in the acquisition of modified functions. Tetrahedral DNA (TD) nanocages have shown the potential to induce the proliferation and differentiation of stem cells when exposed to different populations of these cells.^54,58–61^ DNA structures can be functionalized with different functional molecules, such as polymers,^62–64^ targeting ligands or therapeutic nucleic acids,^65,66^ peptides,^56,57,67,68^ and proteins,^69,70^ with precise control over valency and orientation.

DNA nanostructures have the potential to be used as multipurpose theranostic agents. However, several challenges need to be addressed before they can be used in the pharmaceutical market. These challenges include *in vivo* stability,^71^ distribution,^72^ cellular uptake,^71^ and the cost of the synthesis.^73^ To overcome these challenges, cautious and systematic studies on the fate and interaction of DNA nanostructures with biological components are essential. It is crucial to determine whether DNA nanostructures remain structurally intact within physiological conditions, resist nuclease degradation, and are fully biocompatible. Strategies are also required to overcome the challenges associated with nucleic acids *in vivo* delivery, such as poor distribution and low cellular uptake. The standardization of methodologies is necessary to thoroughly characterize the interaction of DNA structures with biological systems, which will accelerate their development for medical applications. In this review, we have summarized the current application in the physical and biomedical areas and highlighted the key bottlenecks and limitations and the future outlook for their better and safer interface with biological systems.

## Structural DNA nanotechnology

Different DNA nanostructures have been developed based on the property of molecular recognition, creating artificial DNA structures. Multiple ssDNA segments hybridize, forming an assembled DNA nanostructure called bottom-up assembly.^74,75^ The construction of the desired built DNA structures from the large-size DNA is called the top-down approach. The “tile-based” assembly of DNA is essential and most important for the evolution of structural DNA nanotechnology. The basic structures of DNA are Holliday junction to double crossover (DX),^76^ triple crossover (TX),^77^ paranemic crossover (PX),^78^ paranemic crossover with two juxtaposed sites (JX2),^78^ and six-helix bundles (6HB)^79^ structures of various dimensions. The term “DNA origami” was introduced in 2006 by an American scientist, Rothemund, at the California Institute of Technology.^74,80^ The molecular self-folding of the long ssDNA into organized DNA scaffold structures is called one-pot assembly. Without the use of any ligation enzymes and restriction enzymes, the Watson–Crick base pairing of the ds-DNA hybridizes. DNA staples are introduced in long ss-DNA, forming well-defined DNA origami structures. Highly complex 2D and 3D DNA nanostructures are easily developed due to their addressability, programmability, and base pairing. Excellent biocompatibility and biostability make DNA origami a potent tool for biological application.^81^ Using this DNA origami technique, different structures were created using M13 bacteriophage genome DNA as a scaffold by caDNAno^13^ software. Researchers have also created a 3D cuboid box using DNA staples from the M13 viral genome.^82^ Six origami regions form a box that can be opened by one lid region. This lock and key concept can be very useful for cargo delivery. Anderson *et al.* have created dolphin-shaped DNA origami using M13 viral DNA.^83^ This demonstrates the potent capacity of DNA origami structures for complex structure design. For the customized construction of DNA origami, the CanDo computational tool was developed in 2011 for 3D modeling, predicting flexibility, and pointing out conditions for maintaining structural integrity.^84^

**Fig. 1:**
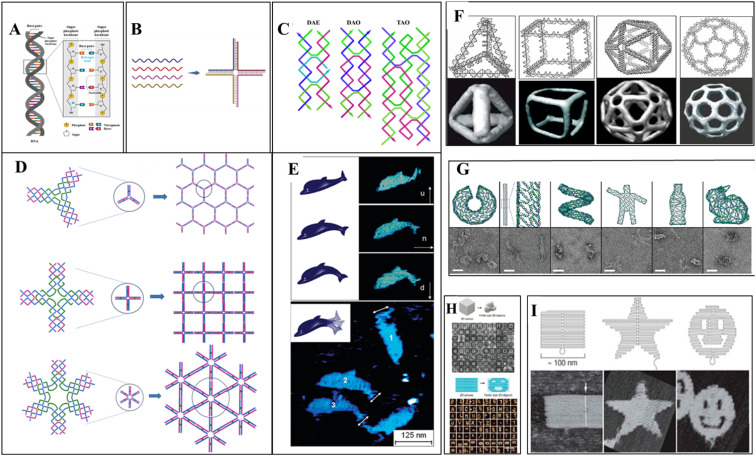
DNA nanostructures: (A) a double-stranded DNA structure showing hydrogen bonding between two strands. (B) Immobile holliday junction: four single-stranded DNA hybridize based on the sequence complementarity, forming a four-way junction. (C) 2D assembled DNA motifs: double crossover structures, DAE and DAO, and triple crossover DNA (four oligonucleotides chain hybridized into three double-helices) structure. DAE and DAO adapted from ref. 87 with permission. Copyright © 1993, American Chemical Society. TAO Adapted from ref. 77. With permission. Copyright © 2000, American Chemical Society. (D) 2D self-assembly of DNA star motifs: three-point-star DNA motif assembled forming a two-dimensional hexagonal nanostructure. Adapted from ref. 88. With permission. Copyright © 2005, American Chemical Society. Cross-shaped DNA self-assembly and a 2D array of DNA motif,^90^ six-point-star DNA motif, and a 2D array of motif. Adapted from ref. 89 with permission. Copyright © 2006, American Chemical Society. (E) Dolphin-shaped DNA origami structure: AFM images of the dolphin-shaped DNA origami with different conformational states. Arrows indicate the orientation of the tail as up (u), normal (n), and down (d). Adapted from ref. 83 with permission. Copyright © 2008, American Chemical Society. (F) 3D DNA origami structures: different DNA nanocages constructed as simple wireframe objects with duplex or DX-based edges (left to right: tetrahedron, cube, icosahedron, and buckyball). (G) 3D DNA origami-based motifs; long viral ssDNA folded using DNA staples into DNA origami structures. (H) ssDNA can form mega-Dalton structures as a tile to assemble with other tiles in either 2D or 3D. (I) 2D origami designs from long ssDNA folded by DNA staples. (F–I) reprinted from ref. 96 with permission. Copyright © 2020, American Chemical Society.

Two-dimensional DNA origami structures were also developed after 2006. Instead of conventional staple DNA, rectangular DNA tiles were used.^85^ Different shapes of DNA tiles were used to create various 2D DNA origami.^86^ The simplest DNA 2D nanostructures are DNA double crossover tiles.^87^ These crossover tiles can be parallel or antiparallel. Triple crossover tiles^77^ are three double helices joined together to form four DNA strands, followed by three-point star-like motif,^88^ six-pointed star-like motif,^89^ cross-shaped motif,^90^ DNA nanotracks with ds-DNA bridges,^91^ and tile-based double-decker motif.^92^ The tile-based self-assembly of the DNA scaffold uses the sticky-end hybridization method, which can create diverse two-dimensional nanostructures. Three-dimensional DNA nanostructures were the new scope of DNA origami. One method for 3D nanostructure designing is using DNA staples; single-stranded long DNA can be organized into well-defined nanostructures. Another technique uses short DNA sequences of about 150 bp to create the desired nanostructure. Automated computer software was widely used for 3D DNA origami.^13^ The tensegrity triangle was the first motif-based 3D DNA structure;^93^ 3D honeycomb structures were created using caDNAno software.^13^ Rothemund *et al.* have made different nanoscale shapes and patterns by folding the DNA (*e.g.*, rectangle, triangle, triangle with trapezoidal domains, triangle with rectangular domains, star, and a wide range of smiley faces).^25^ Linuma *et al.* have developed different DNA polyhedrals (tetrahedrons, cubes, triangular, pentagonal, and hexagonal prism) from megadalton monomers using DNA tripods.^94^ Different 3D geometry shapes were designed from DNA polyhedral meshes, and computer-aided DNA structure development made it easy to render the DNA.^95^ Some representative DNA nanostructures are shown in Fig. 1.

## Biological application and limitations of DNA nanotechnology

DNA nanostructures play a crucial role in various biological applications, encompassing tissue engineering, cancer therapy, bioimaging, drug delivery, and diagnosis. The versatility and programmability of DNA molecules have enabled their utilization in diverse biomedical fields. This review section aims to delve into the manifold applications of DNA nanostructures and shed light on the associated challenges.

### 1. Tissue engineering

Tissue engineering is an innovative field that explores using cells and appropriate physical and biochemical elements, individually or in combination, to facilitate the formation of structures resembling natural tissues. Tissue engineering encompasses various areas of focus, including the construction of cartilage and bone, engineering of vascular tissues, regeneration of nerve tissues, development of skin tissues, advancements in oral tissue engineering, creation of tendon and ligament tissues, corneal tissue engineering, as well as the engineering of vital organs such as the liver, pancreas, kidney, and lung.^4,97^ DNA nanotechnology has become a promising tool in tissue engineering, and scientists have used various techniques to realize its promise fully. These methods include various techniques and approaches to create functioning tissue constructions with improved qualities and capacities. DNA nanostructures are essential for building intricate three-dimensional (3D) scaffolds in tissue engineering. These scaffolds replicate the natural extracellular matrix and act as a template for cell development and organization.

**Fig. 2:**
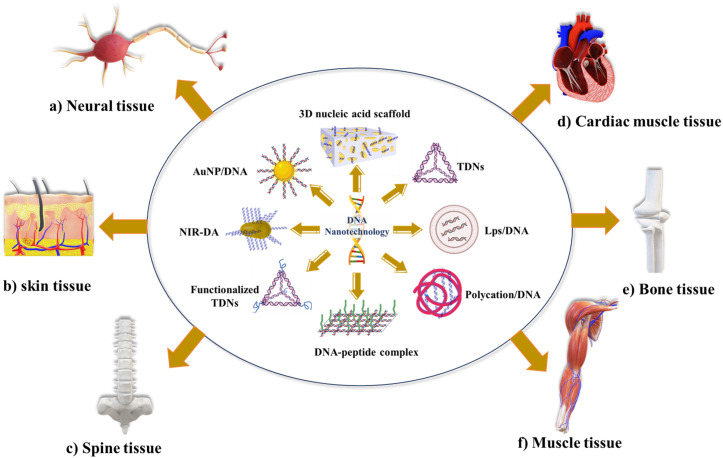
The biological application of DNA nanotechnology in tissue engineering: (a) biological application of different DNA nanomaterials in neural tissue engineering (*e.g.*, TDNs, NPs with pDNA, and polycation with pDNA). (b) The biological application of DNA nanomaterials in skin tissue engineering (*e.g.*, TDNs and polycation/pDNA). (c) The biological application of DNA-based materials in spine tissue engineering. (d) The biological applications of DNA nanomaterials in cardiac tissue engineering (*e.g.*, nanofibrous loaded with pDNA and TDNs). (e) The biological applications of DNA nanomaterials in bone tissue engineering (*e.g.*, TDNs, lipopolysaccharide nanoparticles with DNA, polypeptide-DNA complex, and polycation/pDNA). (f) The biological applications of DNA nanomaterials in muscle tissue engineering (*e.g.*, near-infrared light-activated DNA agonist (NIR-DA) and nanoparticles with DNA).

In bone tissue engineering, the pDNA delivery system was first designed, which transfers the bone morphogenetic protein, fibroblast growth factor, and vascular endothelial growth factor.^4,51,52^ The scaffold-mediated transfer of pDNA, combined with poly(lactic-*co*-glycolic acid)/Hap, was designed to transfer bone morphogenetic protein gene.^98^ Qiao *et al.* encapsulated BMP-2/PEI (polyethyleneimine) nanoparticles with PLGA to deliver BMP-2 *in vitro* for bone formation.^53^ Many other materials are used to deliver pDNA to enhance the delivery and bone regeneration.^99–101^ Tetrahedral DNA nanostructures (TDNs) were also employed; TDNs promoted chondrocyte proliferation and phenotype.^102^ Sirong *et al.* used TDNs with wogonin to inhibit inflammation and promote chondrocyte proliferation to slow or reverse the progression of osteoarthritis.^103^ Zhou *et al.* showed that DNA tetrahedron nanostructures could promote proliferation and osteo/odontogenic differentiation of dental pulp stem cells.^104^ Ma *et al.* developed folic acid-modified triangular DNA origami nanostructures (FA-tDONs) for the targeted therapy of rheumatoid arthritis (RA).^105^ The advantages of ROS- and nitric oxide (NO)-scavenging capability of DNA, FA-tDONs target ligands for pro-inflammatory M1 macrophages, which predominantly promotes RA progression, including monocyte recruitment, fibroblast proliferation, and proinflammatory cytokine secretion. It also facilitates the M1-to-M2 transition of macrophages, an effective alleviation of inflammatory damage, and attenuates rheumatoid arthritis (RA). Son *et al.* have reported DNA aptamer-conjugated hydroxyapatite that promotes bone regeneration and angiogenesis.^106^ Recently, Athanasiadou *et al.* designed DNA hydrogels that promote bone repair.^50^ With the advancement in recent decades, different materials were developed, from pDNA with complex materials to TDNs with bioactive material to enhance the stability and poor penetration. However, the utilization of complex materials in cells or organs presents a primary challenge related to cellular uptake through endocytosis, and physiological conditions and material surface properties affect cellular uptake.^4,33^Fig. 2 represents applications of DNA nanotechnology in tissue engineering.

In neural tissue engineering, regenerative medicine proposes that the utilization of biomaterials and tissue engineering techniques holds promise for the restoration of damage in the nervous system. In particular, tissue engineering methods have been developed to facilitate the delivery of cells and signals, such as neurotrophic factors, to promote nerve regeneration.^55^ Stem cell-based therapy is a commonly employed approach; however, effective differentiation and proliferation following transplantation pose challenges in neural stem cell-based therapy. Consequently, there has been significant research investigating the potential of tetrahedral DNA nanostructures (TDNs) as DNA-based nano-biomaterials to enhance cell proliferation and differentiation. TDNs have garnered extensive attention due to their capacity to promote these cellular processes and offer promising prospects for improving neural stem cell-based therapeutic strategies. *In vitro* studies have demonstrated that tetrahedral DNA nanostructures (TDNs) effectively promote the proliferation of NE-4C stem cells by activating the Wnt/β-catenin pathway and differentiation by inhibiting the notch signaling pathway.^107^ The addition of small peptides with DNA nanostructures can also induce the differentiation and proliferation of neural stem cells.^56,57^ Many other biodegradable neural scaffolds were also used.^4,108^ Despite the emergence of numerous neural scaffolds, the effectiveness of these biomaterials in bringing about beneficial changes and complete integration with surrounding tissues as comprehensive therapies remains limited. Drug therapy has been utilizing neurotrophic growth factors (NTFs) to guide healthy regeneration and anti-inflammatory agents to mitigate the damaging effects of inflammation. However, these molecules face challenges related to inadequate pharmacokinetic properties, low solubility, potential harm to non-target cells and tissues, and difficulty crossing the blood–brain barrier.^32^ The main concern lies in the reproducibility of *in vitro* results into *in vivo* results.^109^

Skeletal muscle tissue comprises 45% of body mass; minor injuries in muscle can be repaired by regeneration, while severe conditions result in irreversible loss of muscle function, and the treatment used was the transplantation of *ex vivo*-cultured muscle cells, which did not show success.^34^ Recently, an innovative nanodevice known as Near-Infrared Light-Activated DNA-Agonist (NIR-DA) has been created by researchers to allow the non-genetic modification of cell signaling and phenotypic in deep tissues. A DNA agonist is coupled to gold nanorods in this nanodevice. Due to the photothermal action, it releases and activates the DNA agonist when exposed to near-infrared light. To start downstream signal transduction in living cells, the activated DNA agonist helps DNA-modified receptor tyrosine kinases (RTKs) to dimerize on cell surfaces. The inducement of RTK signaling by NIR-DA enables the regulation of cytoskeletal remodeling, cell polarization, and directional migration. *In vivo* experiments show that the NIR-DA system has the potential to mediate RTK signaling, skeletal muscle satellite cell migration, and myogenesis, which has interesting implications in skeletal muscle regeneration.^110^ Blumenfeld *et al.* reported magnetic nanoparticles with genomic DNA, which can rescue cultured cardiac myoblasts. These genomic DNA-coated magnetic nanoparticles capture some chemotherapeutic agents (doxorubicin, cisplatin, epirubicin) from human serum.^111^ Zhang *et al.* used tetrahedral DNA nanostructures (TDNs) for myocardial ischemia-reperfusion injury (MIRI). The anti-inflammatory/antioxidative properties of TDNs make them therapeutic candidates. TDNs effectively reduce oxidative damage and apoptosis by suppressing ROS overexpression and modulating apoptosis-related proteins, protecting cardiomyocytes from oxidative stress, which can surely induce MIRI.^112^ The use of DNA-based biomaterial in cardiac muscle tissue engineering is limited, and biosafety and biocompatibility are the main questions.

DNA nanotechnology has potential for other tissue engineering; TDNs were also used for skin tissue engineering and drug delivery into the subcutaneous tumor.^43^ Currently, many biomaterials for the promotion of wound healing and skin regeneration have been developed.^113,114^ Guo *et al.* used DNA nanomaterial for the therapy of full-thickness burn wounds.^115,116^ Vascular endothelial growth factor-165 (VEGF-165)/*N*,*N*,*N*-trimethyl chitosan chloride (TMC) complexes were encoded by pDNA and inserted into a bilayer porous collagen-chitosan/silicone membrane dermal equivalents (BDEs). These types of pDNA-based delivery systems could promote skin regeneration. Similarly, a framework nucleic acid-based transdermal delivery system was also developed; in the mouse melanoma model, framework nucleic acid (TDNs) with doxorubicin was delivered to the tumor site subcutaneously.^117^ Zhu *et al.* also reported that TDNs can promote fibroblasts and keratinocyte proliferation and migration. By activating the AKT-signaling pathway, TDNs decreased the production of interleukin-1 beta (IL-1β) and tumor necrosis factor-alpha (TNF-α) in HaCaT cells and boosted the secretion of vascular endothelial growth factor (VEGF) and basic fibroblast growth factor (bFGF) in HSF cells.^118^ Thus, TDNs could be used to deliver such agents to the skin tumor because of the penetration efficiency and bioactivity. However, skin penetration of the different DNA structures is highly dependent on the shape and size of the DNA nanostructures.

Other than tissue regeneration, DNA nanostructures were widely used to enhance angiogenesis,^106,119,120^ which eventually will help in tissue engineering. Researchers have also used DNA origami nanostructures with rectangular, triangular, and tubular shapes for the treatment of acute kidney injury (AKI). Rectangular DNA origami nanostructure (DON) has shown higher efficacy *in vivo*, similar to the antioxidant *N*-acetylcysteine. These rectangular DON specifically accumulate in the kidney. This can be an effective therapeutic for kidney-related diseases.^72^

### 2. Cellular uptake and drug delivery

DNA nanotechnology is a promising approach for designing self-assembling nanostructures that can be used for drug delivery. Drug delivery faces several challenges, including degradation, solubility, toxicity, and other biological side effects of drug molecules, as well as piercing *in vivo* barriers such as cellular membranes and the blood–brain barrier.^41^ However, nanoparticles smaller than 15 nm can cross this barrier.^121^ Cellular uptake is a crucial factor in the application of DNA nanostructures in biomedicine, particularly in drug delivery and targeted therapy. DNA strands are hydrophilic and negatively charged, which makes it difficult for mammalian cells to take them up. However, assembling DNA into 3D structures can trigger binding to specific receptors and cause cellular uptake.^71^ The wireframe tetrahedron is the most studied DNA structure, and several papers have reported its cellular uptake.^122–124^ Researchers discovered that the tetrahedron uses a “corner-attack” mechanism to penetrate the cell by minimizing contact with the cytoplasmic membrane.^125^ Nanoparticles have different properties, such as size, shape, and material, which are crucial in their distribution in the body. The efficiency and process of cellular uptake depend on the cell types and the physicochemical properties of the complex materials, such as the surfaces of cells and materials and the shape and size of materials.^126,127^ Similar geometries with different internal designs can also impact cellular uptake and penetration. A wireframe design of a DNA origami rod has higher penetration than a lattice-based origami DNA rod in cell and cell spheroid tissue models.^128^ As shown in previous applications, different chemotherapeutic agents, genes, and growth factors have widely been delivered using DNA nanostructures. For instance, Jiang *et al.* used DNA origami for the treatment of acute kidney injury (AKI).^72^ Engelen *et al.* reported that the multilayer icosahedral origami shell was achieved using 20 identical triangle origami units and shape-complementary stacking. Bivalent binding sites were employed to build IgG antibody–antigens bridges using adjacent triangular units that had antigen pairs on their triangle–rigger borders. Once soluble antigen molecules were incubated, the structural reconfiguration was initiated, and the IgG staples were replaced from the icosahedral shell. With DNA origami, a new nanocarrier for stimuli-responsive cargo release, this work clarified a sound antigen-sensing technique.^129^ DNA origami-based “nanorobots” encapsulated with the antibodies against the antigen were expressed on the surface of leukemia. These highly organized DNA nanorobots are capable of transporting molecular payloads to cells, sensing cell surface inputs for conditional and triggered activation, and changing their structure for the delivery of the payload.^130^ Arnon *et al.* used this type of DNA robot in a living host.^131^ This could be used to perform many biological activities. The Ding research group has reported many DNA origami-based devices for therapeutics delivery. An autonomous tubular DNA nanodevice with doxorubicin and siRNA was designed to target cancer precisely.^132^ DNA nanodevice-based vaccine was also developed by this group.^133^ Other DNA origami-based drug delivery vehicles with therapeutic effects were also developed in the past decade.^133–136^ A recent study used drug affinity responsive target stability (DARTS) with liquid chromatography/tandem mass spectrometry (LC-MS/MS) techniques to explore the endocytosis process of TDNs. The study reported that the endocytosis of TDNs was related to caveolin-1 (CAV1) and micropinocytosis-related protein sorting nexin5 (SNX5). However, the cellular internalization processes of other DNA nanomaterials are not clear to date, which needs more exploration.

Functionalizing DNA nanostructures can enhance their cellular uptake, which is important for their use as therapeutic delivery vehicles. Different functionalization strategies can be used, such as cationic lipid modification,^123^ targeting ligands,^48,137^ endosomal escape moieties,^138^ surface charge modification,^139^ and size and shape optimization.^126^ DNA nanostructures can be functionalized with appropriate ligands to improve the targeted delivery of drugs. For example, DNA nanomaterial (TDN) modified with Toll-like receptor 4 (TLR4) small interfering RNA (siRNA) on each vertex (TDN-TLR4-4siR) prevents bisphosphonate-related osteonecrosis of the jaws (BRONJ). This nanomaterial aims to regulate mitochondrial homeostasis in polarized macrophages, considered a critical pathogenic mechanism of BRONJ.^140^ DNA tetrahedron functionalized with typhaneoside results in a dual-targeted complex. It is designed to target mitochondria and renal tubules, providing increased antiapoptotic and antioxidative effects, as well as promoting mitochondria and kidney function restoration.^141^ Functionalizing TDNs with unmethylated CpG motifs can induce immunostimulatory effects and produce pro-inflammatory cytokines.^142,143^ Aptamer-DOX conjugates have been designed to target prostate-specific membrane antigen (PSMA), which upon binding, leads to internalization and drug release inside the cell.^38^ DNA origami-loaded DOX has also been shown to exhibit efficacy.^44^ DNA buckyball with nucleoside analog reduces tumor growth.^144^ Chemical modification facilitates designing stimuli-responsive drug delivery systems for controlled drug release,^48,145^ including light-responsive photoisomerization,^146,147^ photocleavage,^148,149^ and photothermal,^110,150^ pH-responsive,^144,151^ glutathione responsive,^145,152^ ATP-responsive,^153,154^ temperature-responsive drug delivery,^155,156^ metal-ion responsive,^157,158^ and antigen-responsive drug delivery.^159^ A pH-dependent controlled drug release for killing cancer epithelial cells has been demonstrated using aptamer conjugated six-point-star DNA motif constructing DNA icosahedron, which carries Doxorubicin.^160^ Recently, light-controlled DNA nanodevice systems were developed for controlled drug release, such as an NIR light-controlled DNA nanodevice, with the GC-rich DNA duplexes and poly(ethylene glycol) (PEG) on the surface of upconversion nanoparticles (UCNPs). The UCNPs release UV light in response to NIR light, which can cause the photolysis of the GC-rich DNA duplexes and cause Dox release with precise spatiotemporal control.^161^ However, there are many challenges in stimuli-responsive DNA nanostructures, such as structural stability, poor permeability, and endosomal escape, as reviewed by Wang *et al.*^145^ Photoresponsive systems face challenges in light wavelength as the UV/visible range is not able to penetrate the tissue; thus, it needs to be in the infrared region.^145^

Self-assembled DNA tetrahedron nanostructures are a promising option for drug delivery due to their excellent biocompatibility, stability, and cellular uptake rates. TDNs have been reviewed for different applications, including drug delivery, antisense oligonucleotide delivery, anti-inflammation, anti-apoptosis, and antioxidation properties.^4^ A DNA tetrahedron delivery vehicle with an active targeting strategy can help achieve stable drug encapsulation and on-demand release in response to the stimuli. This innovation can be used to create a versatile platform for various drug delivery needs.^162^ The tetrahedral DNA nanostructure (TDN) has already demonstrated its value in delivering different bioactive molecules due to its excellent biocompatibility, programmability, and remarkable cell and tissue permeability. TDNs with different therapeutic molecules like Doxorubicin,^122,163^ 5-Fluorouracil (5-FU),^164^ temozolomide (TMZ),^165^ and MB^166^ have been widely used with different aptamers for cancer therapies. However, drug delivery by systemic administration is still challenging since several barriers must be removed before the nanoparticles can release their cargo specifically to the specific molecular target. Nanoparticles must effectively penetrate the tissue in the targeted area while escaping clearance by liver and spleen macrophages.^73^ One significant area for improvement is the knowledge of the mechanism of DNA nanomaterial and cell interaction and poor tissue aggregation. The main barrier for efficient drug delivery or cellular uptake is crossing the cellular membrane and the intracellular protections, which affects the dynamics of self-assembled DNA nanostructures.^71,126^ However, modification in the DNA nanostructure can enhance cellular uptake. Ji *et al.* developed a DNA nanotube-peptide complex with concatenated DNA strands, locked DNA strands, and a cell-penetrating peptide as a drug delivery system for cancer and imaging agents.^167^ In a recent study, TDNs with the modification of cationic lipids enhanced the cellular uptake.^123^ Another recent study has shown that DNA origami pores in the liposomes can be useful for drug delivery.^168^ DNA origami pores can permit the diffusion of small molecules as well as larger proteins.

Another vital aspect is that the precise release of the drug into the target is essential. DNA nanostructures are mainly used as vehicles for drugs to prevent drug leakage, and DNA carriers should be stable enough.^71^ Many research groups have successfully used DNA vehicles for drug release.^44,169^ Combining active cellular targeting with stimuli-responsive drug release can improve the drug efficacy and diminish off-target effects in biological systems. DNA nanostructures have potential as drug delivery vehicles, highly specific diagnostic devices, and tools to decipher how biomolecules dynamically change their shapes and interact with each other and with the candidate drugs. Recently, Madhanagopal *et al.* reported that caffeine can deintercalate small molecules from double-stranded DNA. This strategy could be used to induce drug release from the DNA nanocarrier.^170^ However, more research is needed to determine crucial details about possible biomedical applications of DNA nanoagents, such as their circulation half-life, pharmacokinetics, size- and shape-dependent properties for passive tumor targeting, uptake, intracellular fate, and clearance process.

### 3. *In vivo* stability of DNA nanostructures

**Fig. 3:**
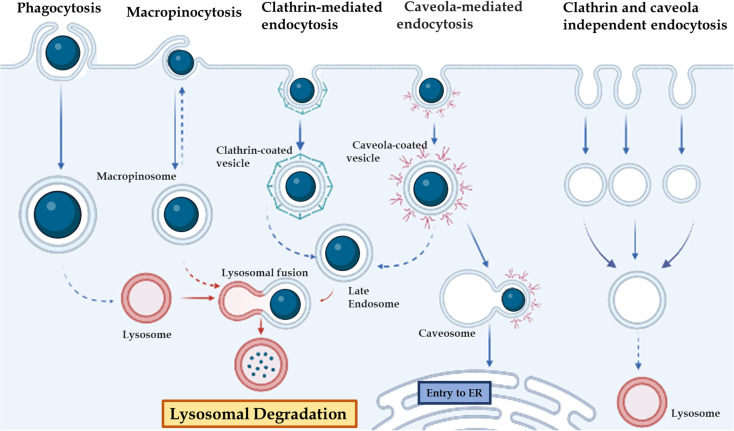
Different gateways for cell entry: phagocytosis, micropinocytosis, clathrin-mediated endocytosis, caveola-mediated endocytosis, and clathrin-caveola independent endocytosis.

The *in vivo* stability of DNA nanostructures can be influenced by various factors, including the chemical nature of DNA, the presence of nucleases, environmental conditions, serum stability, size and shape, and immunogenicity.^71,96,171^ The stability of DNA nanostructures can vary depending on the environment, such as elevated temperature,^172^ pH,^171,172^ high-salt and low-salt conditions, nucleases,^173^ and the presence of chaotropic agents and crowding agents. Stability in the physiological condition is essential with a change in the state of the self-assembly environment and physiological environment. A major challenge to the application of DNA-based nanostructures is their susceptibility to attack by a variety of nucleases present in body fluids such as blood, urine, and saliva. Although nucleases have important biological functions, they also interfere with the function of DNA-based structures in biological applications, necessitating the study of DNA nanostructure biostability *in vivo* or in conditions that mimic body fluids. For practical applications, DNA nanostructures need to meet certain biostability thresholds. For example, in biosensing, DNA nanodevices must remain intact when mixed with samples (typically, biofluids such as blood, serum, and urine), as unpredictable and sudden degradation can lead to false signals. In drug delivery, the biostability of DNA nanostructures is important to protect the drug from harsh physiological conditions until it reaches the destination site in the body Fig. 3.

### 4. Diagnosis and biosensing

Controlling the progression of the disease and raising patient survival rates depend heavily on early diagnosis, which gives crucial information for prompt management.^46^ The development of biosensors employing DNA nanotechnology is growing, and this technology has numerous advantages over the currently used diagnostic techniques.^47^ The use of DNA nanostructure in biosensing is one of the significant applications. In addition to nucleotide sequences, ion concentrations, pH, and peptides, DNA sequences can react with various outside stimuli. These response mechanisms provide biosensors with their chemical foundation and enable the detection of external surroundings.^174^ DNA nanostructures conjugated with other nanoparticles can create colorimetric detection techniques.^175,176^ Shiu *et al.* developed an aptamer-based detection system for malarial diagnosis; DNA tetrahedrons and other DNA structures like square, pentagon-based pyramids, and prisms were used to detect molecular targets. This system distinguishes *Plasmodium falciparum* lactate dehydrogenase (PfLDH) from human lactate dehydrogenase.^177^ By identifying the intracellular target miRNA21 and the cellular membrane target nucleolin, Zhang *et al.* developed an effective cancer detection system, a tetrahedral framework DNA-enhanced (TDN-enhanced) HCR detection system (T-probe system).^178^ Recently, a biosensing and detecting platform was developed to detect mucin 1 (MUC1) and nucleolin overexpressed in breast cancer, a dual-responsive DNA tetrahedron nanomachine (drDT-NM) with two aptamers attached, MUC1 aptamer (MA), and a hairpin H1* encoding nucleolin-specific G-rich AS1411 aptamer. When the bivariate MUC1 and nucleolin are bound explicitly by drDT-NM, two separate hybridization chain reactions as amplification modules are started. The HCR of MA has the quencher BHQ1 and fluorescein acting in tandem to sense MUC1. Nucleolin responsiveness is achieved by operating the HCR of AS411 aptamer with two additional hairpins programmed with two pairs of AS1411 splits. The parent AS1411 aptamers are collaboratively merged and folded into G-quadruplex concatemers in the shared HCR duplex products to embed Zn-protoporphyrin IX (ZnPPIX/G4) for fluorescence signaling readout, resulting in a susceptible intracellular test and observable cell imaging.^179^ Recently, an ultrasensitive and electrochemical biosensor was developed with DNA nanotubes to detect cancer-related biomarkers microRNA-21 (miRNA-21) and glutathione (GSH).^180^

**Fig. 4:**
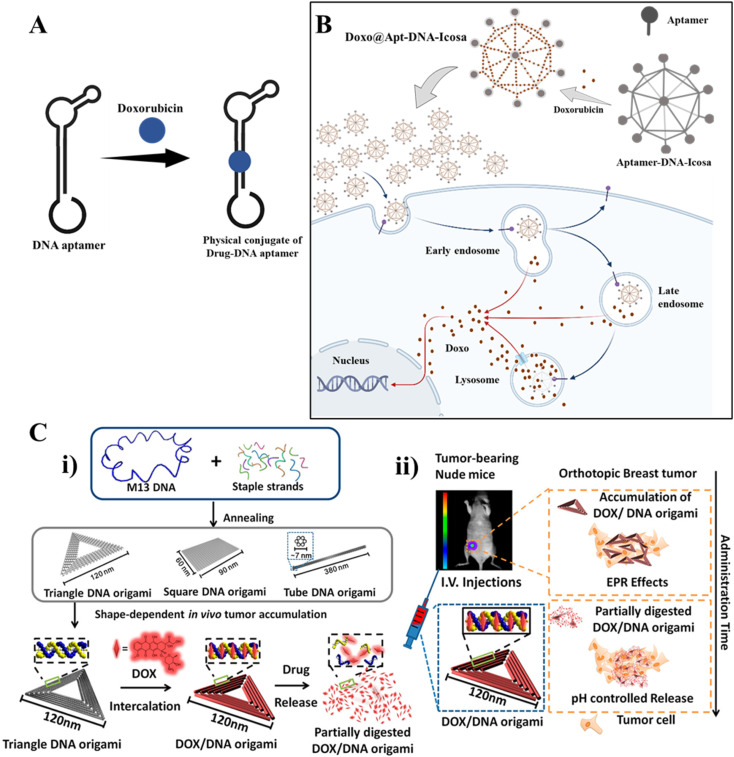
DNA nanotechnology for drug delivery: (A) DNA aptamer-drug physical conjugate; a DNA aptamer intercalates with a drug forming a drug-aptamer conjugate. (B) Drug delivery using a DNA nanostructure conjugated with an aptamer: DNA icosahedron formed from a six-pointed star motif conjugated with an aptamer for loading doxorubicin. Adapted from ref. 160 with permission. Copyright 2011 American Chemical Society. (C) DNA carrier–drug complex. (i) Long viral ssDNA scaffold (M13mp18 viral genomic DNA, blue) hybridizes with rationally designed DNA staples, which can fold into different shapes (triangular, square, and tube origami shapes). A breast tumor model was used to investigate the biodistribution of unstructured M13 DNA and different DNA origami nanostructures. The triangle-shaped DNA origami demonstrated optimal tumor accumulation *in vivo*; it was then used for doxorubicin intercalation. The Watson–Crick base pairs in the double helices of DNA origami serve as docking sites for doxorubicin intercalation (DOX/DNA origami, red). (ii) Tail-injected DOX/DNA origami complexes were transported *via* blood circulation and owing to EPR effects and were accumulated in nude mouse breast tumours. Adapted with permission from ref. 135. Copyright 2014 American Chemical Society.

Dong *et al.* have reviewed DNA nanomachines for cancer diagnosis, biomarkers such as circulating tumor cells (CTCs), exosomes, and circulating tumor DNA in body fluids, and mRNAs, miRNAs, mutated proteins, and telomerase are intracellularly targeted for the diagnosis.^181^ But, early cancer detection is a significant challenge in DNA nanotechnology-based cancer diagnostics. When cancer first develops, the biomarkers needed for biomarker-based detection may be minimal. Some diagnostic methods are often unsuitable for on-site analysis where rapid diagnosis is required due to expensive equipment and limited resources. Fig. 4 represents DNA nanostructure conjugates as drug delivery systems in different targeted sites.

### 5. DNA technology for cell surface engineering

The cell membrane is a dynamic organelle that acts as a bridge between the cell and its environment, vital in facilitating communication between the two. Molecular manipulation of the cell membrane can revolutionize our comprehension of cellular processes, redefine cellular functionality, and contribute to developing new tools for regulating cell-environment communication.^182^ One promising approach to cell membrane engineering is synthetic DNA for cell-surface engineering.^183^ DNA offers several advantages over other polymers for constructing functional materials, including its ability to be chemically synthesized with high purity, its capacity to interact with other DNA molecules through Watson–Crick base-pairing rules, and its potential to select DNA aptamers from synthetic libraries with high binding specificities and affinities.^184^ DNA can be immobilized on the cell surface through covalent or noncovalent methods. Covalent conjugation involves modifying amino acids and sugar moieties, while noncovalent methods include hydrophobic insertion and molecular recognition.^185–187^ The primary objective of engineering cell surfaces is to regulate and comprehend cell-environment communication.^183^ This regulation encompasses both the facilitation and inhibition of cell-environment interactions. Notable examples include the promotion of cell–cell recognition, control of signaling pathways, and the safeguarding of therapeutic cells. Additionally, synthetic DNA can be designed as probes on the cell surface to detect the intracellular or extracellular microenvironment as nucleic acids can undergo conformational changes when binding target molecules.^188^

Different types of DNA-based modules have been employed for cell surface engineering, including single-stranded DNA (ssDNA), simple framework nucleic acids (FNAs) such as DNA tetrahedra and cubes, and complex DNA nanostructures like DNA origami and DNA networks. Amphiphilic ssDNA is the simplest DNA-based module used for cell surface engineering,^189^ and researchers have been able to manipulate cell–cell interactions through DNA base-pairing utilizing complementary sequences. To enhance the stability and retention time of ssDNA-based cell surface engineering, various DNA nanostructures have been introduced to the cell surface.^190,191^ For instance, simple FNAs with multiple anchoring sites, such as amphiphilic tetrahedral DNA with a pendant ssDNA at one vertex and three cholesterol tags at the other vertices, have been employed to engineer cell surfaces. These pyramidal DNAs exhibit improved membrane-anchoring stability and higher target accessibility than cholesterol-labeled ssDNA. FNAs also provide multiple anchoring sites for integrating various functional modules, which can implement complex operations on cell surfaces. Similarly, DNA triangular prisms have been utilized to fabricate DNA-based logic gate nanostructures for carrying out Boolean logic operations on the cell surface. This DNA nanostructure consists of two aptamer/cDNA conjugates on the bottom face as recognition modules and three ssDNA on the top face as a computing module. By associating different modules, this DNA nanostructure immobilized on the cell membrane could facilitate the manipulation of complex operations on the cell surface.

### 6. Immunostimulatory effect

**Fig. 5:**
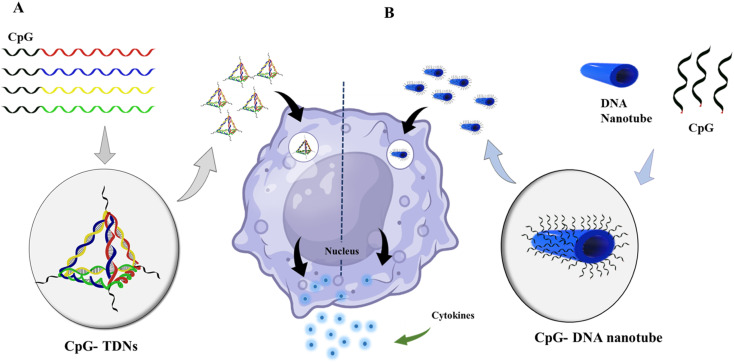
Immunostimulatory effect of DNA nanomaterials: (A) schematic showing the assembly of CpG-bearing DNA tetrahedron and its immunostimulatory effect.^105^ (B) Design of a 30-helix DNA origami tube and endocytic pathway. Different types of CpG-H′s; blue cylinders are double helices DNA nanotubes, and black lines on the tube indicate the possible connection sites for CpG oligonucleotides. DNA origami tube internalized by endocytosis; vesicle segregated by the Golgi apparatus containing the transmembrane toll-like receptor (TLR9); then TLR9 and DNA origami fused with an endosome; recognition of CpG sequence by TLR9 begins the signaling cascade; surface molecules express sand releases cytokines that stimulate further immune response.

DNA nanotechnology has the potential to stimulate the immune system in various applications, such as cancer immunotherapy, vaccine development, and immune modulation. One of the advancements in this field is the development of DNA-launched nanoparticle vaccines, also known as DLnano-vaccines. These vaccines are composed of extremely small particles that display multiple copies of an antigen, similar in size to bacteria and viruses. DLnano-vaccines have been designed to elicit strong immune responses.^192^ DNA nanotechnology enables the design of custom shapes and dynamics as well as site-specific incorporation of diverse guests. This capability has been used to manipulate the signaling pathways that regulate immune responses and engineer cell-surface receptors for cell manipulation. For instance, scientists at The Wistar Institute have successfully delivered nanoparticle antitumor vaccines that stimulated robust CD8 T cell immunity and controlled melanoma growth in preclinical models using synthetic DNA technology.^193^ CpG is a well-known and commonly used immunotherapeutic and vaccine adjuvant. However, it has some disadvantages, such as being unstable and low in efficacy. To overcome these limitations, researchers are exploring the use of nanocarriers to transport CpG and enhance its effectiveness. DNA-based nanocarriers have the potential to be effective in delivering cytidine-guanosine-dinucleotides (CpG) and other immunotherapeutics.^194,195^ In 2011, researchers developed a delivery system that combined a DNA nanotube with CpG oligonucleotides, resulting in immunostimulatory effects. Unmethylated CpG DNA induces adaptive immune responses by triggering powerful T helper (Th)-1-type innate immune responses. This innovative approach can potentially enhance the effectiveness of DNA vaccines and improve immunotherapy for various diseases, including cancer.^196^ Unmethylated CpG DNA induces adaptive immune responses by inducing powerful T helper (Th)-1-type innate immune responses. Induces activation of Th1 cytokines such as interleukin (IL)-12 and interferon (IFN). When it comes to allergies and asthma, Th2-type immune responses are crucial. In this circumstance, CpG DNA can suppress and block Th2 immune responses.^36^ Different DNA nanostructures were reported, with CpG exhibiting immunostimulatory effects.^35,197^ CpG-grafted tetrahedron DNA structure transfected in RAW264.7 cells also induced the upregulation of tumor necrosis factor (TNF)-α, interleukin (IL)-6, and IL-12.^42^ Liu *et al.* demonstrated the *in vivo* immunostimulatory effects of DNA nanostructure using CpG oligonucleotide attached with TDNs and constructed with an adjuvant streptavidin. This antigen-adjuvant vaccination combination was given to mice with immunogenicity and made antibodies against the antigen complex.^198^ Many factors, including long-term immune response, stability, and the design of multispecific vaccination complexes, must be considered when using DNA nanotechnology in vaccine development Fig. 5.

## Future prospects of DNA nanotechnology

DNA nanotechnology has numerous benefits, but there is still a need for significant development in some areas. One crucial aspect is the creation of more effective processes for synthesizing and constructing DNA nanostructures to reduce the real cost of synthesis and increase the yield. The complexity and length of current methodologies prevent them from being scaled up or used in real-world situations. Additionally, it is challenging to improve the stability and toughness of DNA nanostructures under varied physiological circumstances. To fully realize the potential of DNA nanotechnology, ongoing research and innovation are crucial. In drug delivery, the issues are stability, endosomal escape, receptor recognition, poor blood circulation, and poor aggregation in the target tissue. The precise knowledge about the uptake pathway can also help to reduce the degradation rate and help to understand the uptake profile. This can be overcome by stabilizing the DNA nanostructures, which can withstand physiological conditions. Modification or functionalization using biochemical moieties can enhance cellular uptake and reduce nuclease degradation.^123,137,199^ However, the enhancement of cellular uptake can vary from different cell lines, sizes, and shapes of the DNA nanostructure used. But still, this modification can be a promising strategy upon standardization.

Combining active cellular targeting with stimuli-responsive drug release can improve the drug efficacy and diminish off-target effects in biological systems. Trigger-responsive DNA ‘nano suitcases’ encapsulated with a siRNA drug can be a potential tool for many cellular applications.^200^ The sustained release of cargo, using these types of DNA nano switches, can be very useful for targeted and controlled drug delivery applications. Cell surface modification with appropriate ligands can be useful to increase the cellular uptake and targeted delivery. In future research, membrane-bound DNA origami structures could facilitate programmed assembly within the membrane to imitate functional biomolecular complexes, such as an immune synapse. This potential is particularly significant considering the widespread application of DNA origami in protein templating and organization. The addition of the modification at the precise location can be done using computational methods. Another concern related to drug delivery is poor circulation time; adding liposomes or any biological membrane can help enhance the DNA nanostructures' circulation rate. DNA-coated with liposomes is reported in some studies.^201,202^ Further enhancement in drug delivery using DNA nanostructures can be done by combining the DNA nanostructures with other biological molecules. In DNA nanostructures, it is possible to incorporate a variety of ligands, labels for bioimaging, antibodies, hormones, and other substances that could be employed for effective and site-specific medication delivery and release. Peptide-DNA hybrid nanotechnology could also generate new classes of artificial extracellular matrices.^203^ It has recently been shown that *ex vivo* cellular scaffolding on which human cervical cancer cells attach robustly, live, and survive with high migration rates, may be produced using the combination of peptide and DNA nanotechnology.

Computational modeling techniques are essential for solving complex problems in engineering. DNA-based computing can be a promising method to overcome these challenges. In tissue engineering, scaffolds must be designed with specific characteristics to provide the right environment for cell development. Computational methods and 3D bioprinting can be used to create these scaffolds.^204,205^ DNA-based computational tools such as Daedalus,^206^ Adenita,^15^ Catana,^14^ V-helix,^17^ Vivern,^18^ Nupack,^19^ and caDNAno^13^ are used for DNA nanoscale design. Computational methods can also be used for DNA nanostructure design and scaffold design in tissue engineering.^16^ Artificial intelligence (AI) is advancing in various sectors, including healthcare. Researchers are exploring the use of DNA nanostructures in robotics, drug delivery, and cancer treatment.^207^ DNA-based nanorobots are being constructed to study cell processes and apply precise forces at microscopic levels. DNA nanorobots are being developed for bio-medicinal applications like drug delivery and biosensing, and in cancer, AI can help in healthcare by improving design and targeting, synthesizing and analyzing therapeutic nanoparticles, and controlling and tuning cargo release.^208^ DNA nanorobots are being developed for bio-medicinal applications such as drug delivery, biosensing, and cancer treatment.^209,210^

DNA nanotechnology is a burgeoning field, and its scope has expanded beyond what has been covered here. With the high predictability and efficiency of DNA-based materials, DNA nanotechnology has potential applications in various fields such as nanoelectronics, materials science, photonics, environmental monitoring, data storage, and space exploration. Due to the programmability and self-assembly properties of DNA-based materials, they are ideal for creating nanoscale electronic devices such as nanowires, nanotransistors, and nanoturbines, which can be used in various applications such as nanoelectronics and data storage. DNA-based materials can also be used to create photonic devices such as optical switches and sensors, leading to the creation of new and innovative photonic devices. In addition, DNA nanotechnology has potential applications in other fields such as computer science, agriculture, and space exploration. For example, in space exploration, DNA nanotechnology can be used to create new electronic components and high-density memory devices, while in agriculture, DNA-based materials can be used to create new and innovative ways to detect and monitor environmental changes.^211^

## Conclusion

DNA nanotechnology has developed significantly over the past 40 years, producing nucleic acid structures widely used in various biological applications. DNA nanostructures and multivalent drug delivery systems are ideal for building structures of various sizes and shapes due to their structural flexibility, programmability, and multiform customizable DNA-based nanostructures. DNA nanotechnology is a promising technique for creating nanostructures of any shape, which has applications in the delivery of drugs, tissue engineering, cancer therapy, biosensing, diagnostics, and immunotherapy. New challenges and opportunities also appear as DNA nanotechnology research develops. Researchers might look into using DNA nanostructures in robotics applications like sorting and distributing nanoparticles. DNA-based nanorobots are being developed to examine cell processes and apply precise forces at microscopic scales. The development of AI and robotics through DNA nanotechnology has enormous potential. The development of more effective methods for synthesizing and building DNA nanostructures with desired functions under varied physiological conditions can be modulated by incorporating artificial intelligence and robotics.

## Conflicts of interest

We declare no conflict of interest.

## Supplementary Material
